# Genome-guided analysis allows the identification of novel physiological traits in *Trichococcus* species

**DOI:** 10.1186/s12864-019-6410-x

**Published:** 2020-01-08

**Authors:** Nikolaos Strepis, Henry D. Naranjo, Jan Meier-Kolthoff, Markus Göker, Nicole Shapiro, Nikos Kyrpides, Hans-Peter Klenk, Peter J. Schaap, Alfons J. M. Stams, Diana Z. Sousa

**Affiliations:** 1 Laboratory of Microbiology, Wageningen University & Research, Stippeneng 4, 6708 WE Wageningen, The Netherlands; 2 Laboratory of Systems and Synthetic Biology, Wageningen University & Research, Stippeneng 4, 6708 WE Wageningen, The Netherlands; 3 Leibniz Institute DSMZ German Collection of Microorganisms and Cell Cultures, Inhoffenstraße 7B, 38124 Braunschweig, Germany; 4 DOE Joint Genome Institute, 2800 Mitchell Drive 100, CA, Walnut Creek, CA 94598 USA; 5 School of Biology, Newcastle University, Ridley Building 2, Newcastle, NE1 7RU UK; 6 Centre of Biological Engineering, University of Minho, Campus de Gualtar, 4710-057 Braga, Portugal

**Keywords:** Comparative genomics, Protein domains, Halophilic, Psychrophilic, 1,3-propanediol

## Abstract

**Background:**

The genus *Trichococcus* currently contains nine species: *T. flocculiformis*, *T. pasteurii*, *T. palustris*, *T. collinsii*, *T. patagoniensis*, *T. ilyis*, *T. paludicola, T. alkaliphilus*, and *T. shcherbakoviae*. In general, *Trichococcus* species can degrade a wide range of carbohydrates. However, only *T. pasteurii* and a non-characterized strain of *Trichococcus*, strain ES5, have the capacity of converting glycerol to mainly 1,3-propanediol. Comparative genomic analysis of *Trichococcus* species provides the opportunity to further explore the physiological potential and uncover novel properties of this genus.

**Results:**

In this study, a genotype-phenotype comparative analysis of *Trichococcus* strains was performed. The genome of *Trichococcus* strain ES5 was sequenced and included in the comparison with the other nine type strains. Genes encoding functions related to e.g. the utilization of different carbon sources (glycerol, arabinan and alginate), antibiotic resistance, tolerance to low temperature and osmoregulation could be identified in all the sequences analysed. *T. pasteurii* and *Trichococcus* strain ES5 contain a operon with genes encoding necessary enzymes for 1,3-PDO production from glycerol. All the analysed genomes comprise genes encoding for cold shock domains, but only five of the *Trichococcus* species can grow at 0 °C. Protein domains associated to osmoregulation mechanisms are encoded in the genomes of all *Trichococcus* species, except in *T. palustris*, which had a lower resistance to salinity than the other nine studied *Trichococcus* strains.

**Conclusions:**

Genome analysis and comparison of ten *Trichococcus* strains allowed the identification of physiological traits related to substrate utilization and environmental stress resistance (e.g. to cold and salinity). Some substrates were used by single species, e.g. alginate by *T. collinsii* and arabinan by *T. alkaliphilus*. Strain ES5 may represent a subspecies of *Trichococcus flocculiformis* and contrary to the type strain (DSM 2094^T^), is able to grow on glycerol with the production of 1,3-propanediol.

## Background

Type strains of existing *Trichococcus* species have been isolated from diverse and geographically spread ecosystems. Various species derive from waste treatment systems or contaminated sites: *T. flocculiformis* (activated sludge) [1], *T. pasteurii* (septic pit sludge) [2], *T. collinsii* (soil spilled with hydrocarbons) [2], *T. ilyis* (sulfate reducing anaerobic sludge) [3], *T. shcherbakoviae* (sludge from low-temperature anaerobic reactor) [4]; while others were isolated from natural environments: *T. patagoniensis* (guano from penguin, Patagonia) [5], *T. palustris* (swamp, Russia) [2], and *T. paludicola* and *T. alkaliphilus* (high elevation wetland, Tibet) [6].

*Trichococcus* species share a very high 16S rRNA gene sequence identity, in the range of 98–100% [2–4, 6]. This often impairs the taxonomic classification of new strains within this genus on the basis of 16S rRNA gene sequence identity, and therefore whole genome comparison needs to be performed. This was traditionally done by experimental DNA-DNA hybridisation, but nowadays it is also possible to use genomic information to perform digital DNA-DNA hybridisation (dDDH) [7] or average nucleotide identity (ANI) [8] analyses. Availability of genomic information provides also the opportunity for comparing and analysing gene/function diversity among different species. Functional genome analysis on the level of protein domains can be used to infer potential metabolic functions, thereby connecting genotype and physiology [9, 10].

*Trichococcus* species are related to the lactic acid bacteria (LAB), and phylogenetically close to the genera *Carnobacterium* and *Aerococcus* [11]. Described *Trichococcus* species can all grow on glucose, cellobiose, D-mannose, fructose and sucrose [1–6]. However, *T. pasteurii* and *Trichococcus* strain ES5 are the only strains within the genus capable of converting glycerol to mainly 1,3-PDO [12], with comparable product yields to those of other 1,3-PDO producers, such as *Clostridium butyricum* and *Klebsiella pneumoniae* [13, 14]. 1,3-PDO is used as a building block in chemical industry [15], and the discovery of new efficient and resilient biocatalysts for its production are of interest for biotechnological industry. In general, *Trichococcus* species have a broad temperature range for growth (commonly from 4 °C to 40 °C) [1–6]. *T. patagoniensis and T. shcherbakoviae* can grow at negative temperatures and tolerate salinities up to 5% (w/v) NaCl [4, 5], which is also the case for several related *Carnobacterium* species, such as *C. funditum*, *C. alterfunditum* and *C. pleistocenium* [16, 17], but no other *Trichococcus* species.

The objective of this study was to use functional genome analysis, based on encoded protein domains, for identifying novel metabolic traits in *Trichococcus* species. Searches were preferentially directed to find properties that can confer versatility to these species in terms of industrial applications such as, types of substrates used, products formed, and resistance to environmental stress.

## Results

### Comparison of protein domains among *Trichococcus* species

**Fig. 1:**
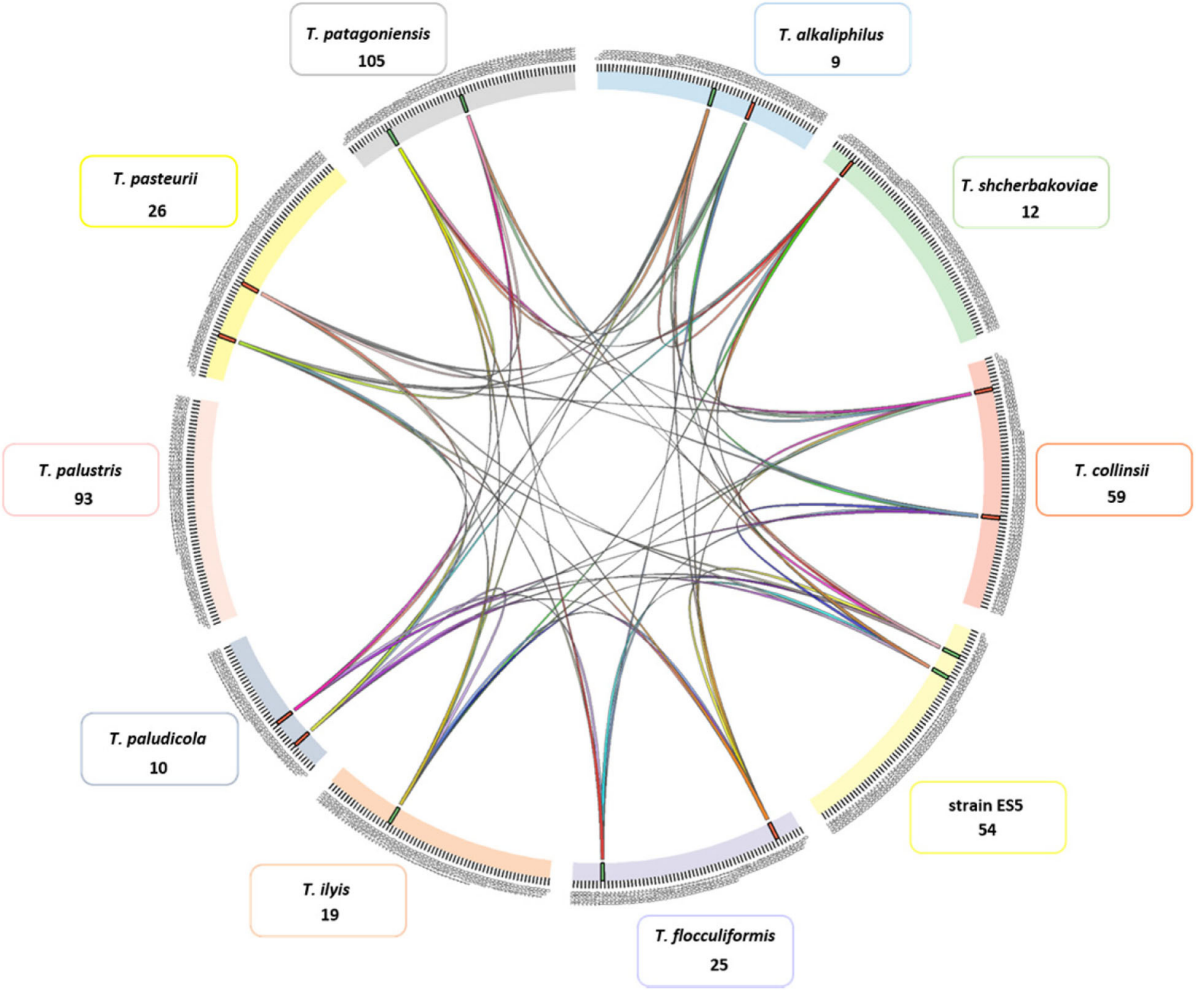
Conserved genomic blocks in the genomes of the ten *Trichococcus* species compared in this study (represented in the figure are only syntenies larger than 45 kb). Each colour represents a *Trichococcus* species and coloured lines indicate shared genomic blocks; The majority of the *Trichococcus* species share two and three 45 kb genomic regions. Note that *T. palustris* has no shared syntenic regions larger than 45 kb with other *Trichococcus* species. Numbers indicated below species names indicate the unique protein domains in each of the genomes

Genome sequences of currently available type strains from the genus *Trichococcus* – i.e. *T. flocculiformis*, *T. pasteurii*, *T. palustris*, *T. collinsii*, *T. patagoniensis*, *T. ilyis*, *T. paludicola*, *T. alkaliphilus*, and *T. shcherbakoviae* were retrieved from NCBI. In addition, we sequenced the genome of *Trichococcus* strain ES5, described by Gelder et al. [12]. Strain ES5 is able to convert glycerol to 1,3-PDO, a property that is also found in *T. pasteurii*, but not in the other *Trichococcus* species. The *Trichococcus* species have similar genome sizes (around 3 Mbp), with the exception of *T. paludicola* that has an estimated genome size of ~ 2 Mbp. However, a completeness assessment of the genomes using BUSCO [18] showed a higher percentage of missing genes in the genome of *T. paludicola* (i.e. 25.1% missing BUSCOs in *T. paludicola* and 2.0–2.7% missing BUSCOs in the genomes of the other *Trichococcus* species) (Additional file 1: Figure S1). Genomes of *Trichococcus* species and other closely related bacteria (Additional file 1: Table S1) were (re) annotated using the pipeline of Semantic Annotation Platform with Provenance (SAPP) [19], which allows to obtain the predicted genes and protein domain annotations. The resulting matrix with all the domains identified in the different *Trichococcus* strains is provided in Additional file 2. Among all the analysed strains (*T. paludicola* was not included in the calculations because of the low number of identified domains), 1424 core protein domains and 1983 pan protein domains could be identified, with multiple protein domains conserved in the different genomes of analysed *Trichococcus* species (Additional file 2). All *Trichococcus* genomes shared genomic blocks of 45 kb, except *T. palustris* (Fig. 1, Additional file 3). In these genomic blocks, 110 domains were identified, with the majority belonging to peptidases, transferases (e.g. acyltransferase, phospholipid/glycerol acyltransferase, phosphatidyltransferase, aminotransferase) and DNA polymerases. Domains of proteins related to carbohydrate metabolism were abundant in the shared genomic blocks among *Trichococcus* species, which correlates to the ability to degrade multiple sugars.

**Fig. 2:**
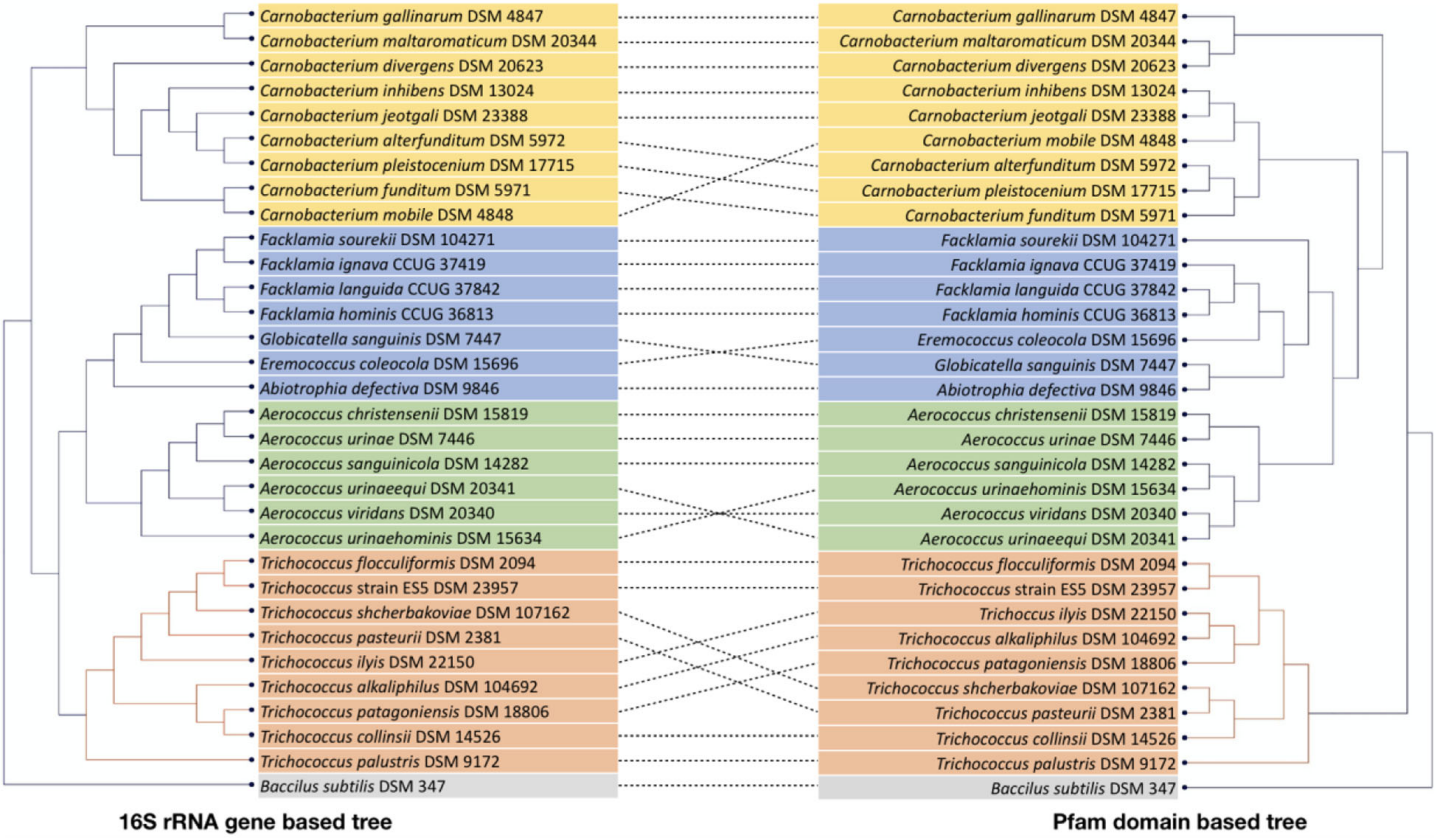
Dendrograms produced by hierarchical clustering of 16S rRNA gene sequences (left pane) and protein domains (right pane), both showing the *Trichococcus* strains analysed in this work and closely related lactic acid bacteria (LAB). *Bacillus subtilis *was used as an outgroup. 16S rRNA gene-based clustering tree was constructed using neighbor-joining algorithm using the software CLC Main Workbench v8.0 (CLC Bio, Aarhus, Denmark). Protein domains are clustered based on presence/ absence in the genomes by applying neighbor-joining method with Dice coefficient using DARwin v6.0 [20]

Protein domain-based clustering of *Trichococcus* species, and other closely related LAB, is shown in Fig. 2 (*T. paludicola* was not included due to the low number of identified domains). Specifically for the *Trichococcus* group, it is patent that using protein domains or 16S rRNA genes results in different clustering of the bacteria. This corroborates with the fact that information in the 16S rRNA gene of *Trichococcus* species is not enough to resolve taxonomy at species level [3, 4, 6], and does not predict the functional relatedness of the different species. 16S rRNA gene and protein domain clustering for the other analysed LAB species is much more conserved (Fig. 2).

The SAPP-generated protein domain matrix (Additional file 2) was mined for the identification of metabolic traits in *Trichococcus* species. A set of metabolic traits (identified in Table 1) was selected for further in vitro testing. One of the most varied aspects among *Trichococcus* species was the capacity to utilize more substrates than previously described, such as glycerol by *T. pasteurii* and *Trichococcus* strain ES5, alginate by *T. collinsii* and arabinan by *T. alkaliphilus* (Table 1). Protein domains related to cold adaption and osmoregulation mechanisms, and to defence mechanisms, were identified in all the analysed *Trichococcus*.

**Table 1:** Genes and protein domains highlighted in this study as a result of functional genome analysis of ten *Trichococcus* strains. Strains (Locus tag_): 1. *T. flocculiformis* (Tflo_)*;* 2*. Trichococcocus* strain ES5 (TES5_); 3. *T. pasteurii* (Tpas_); 4. *T. palustris* (Tpal_); 5. *T. collinsii* (Tcol_); 6. *T. patagoniensis* (Tpat_); 7. *T. ilyis* (TR210_); 8. *T. alkaliphilus* (PXZT_); 9. *T. paludicola* (Ga019_); 10. *T. shcherbakoviae* (TART1_)

Feature	Functional genome annotation	Locus tag
Strain	(Protein domains)	
1,3-PDO production		
2|3	Glycerol kinase (IPR005999, IPR018483, IPR018484, IPR018485)	TES5_2082|Tpas_2911
2|3	Dihydroxyacetone activator (IPR009057, IPR015893)	TES5_2083|Tpas_2912
2|3	Glycerol dehydrogenases (IPR018211, IPR001670)	TES5_2084|Tpas_2913
2|3	Phosphoenolpyruvate phosphotransferase (IPR004006, IPR004007, IPR012736, IPR004701, IPR012844)	TES5_2085–2087|Tpas_2914–2916
2|3	1,3-propanediol dehydrogenases (IPR001670, IPR018211)	TES5_2088|Tpas_2917
2|3	Dihydroxyacetone (IPR004007, IPR012737)	TES5_2089–2090|Tpas_2918–2919
2|3	Glycerol dehydratase (IPR003206, IPR016176, IPR003208, IPR010254, IPR003207	TES5_2091–2093|Tpas_2920–2922
2|3	Glycerol dehydratase activator (IPR028975, IPR030994, IPR003208, IPR010254)	TES5_2094–2095|Tpas_2923–2924
2|3	Cobalamin adenosyltransferase (IPR016030, IPR029499)	TES5_2096|Tpas_2925
2|3	Hypothetical protein (IPR005624)	TES5_2097,2099|Tpas_2926, 2928
2|3	Glycerol uptake facilitator (IPR000425, IPR022357, IPR023271)	TES5_2098|Tpas_2927
Alginate utilisation		
5	Alginate lyase (IPR008929)	Tcol_1369,1377,1704
Arabinan utilisation		
6	Glycosyl hydrolase (IPR033132)	Tpat_54,101,590,610,948-949,136,1167,1171,1259,2028,2033, 2527–2528,2577,2585,2682
6	Metal-dependent hydrolase (IPR032466)	Tpat_57,88,320,321,954,1043,1060,1227,1247,1391, 1392,2241
6	Extracellular endo-alpha-(1- > 5)-L-arabinanase (IPR032291)	Tpat_1197,1296
Psychrotolerance		
All	Cold-shock protein (IPR000194, IPR003593, IPR004665, IPR011112, IPR011113, IPR011129, IPR012340, IPR027417)	TR210_741|Tpas_88|Tpal_285|TES5_627|Tflo_313| Tcol_65|Tpat_494|PXZT01000007.1_99| Ga0192377_1015_35 | TART1_1674
All	Cold shock protein signature (IPR002059, IPR011129, IPR012340, IPR019844)	Tpat_494, 1801, 1802, 1901, 1923|Tcol_65, 532, 554, 1698, 1699|Tflo_313, 455, 458, 688, 980|TES5_627, 827, 849, 1357,1359|Tpal_285,869,1036,1801,1820,1877| Tpas_88,599,1471,1472,2297,2758|TR210_741,1024,1470, 1709, 1819, 1842| PXZT01000016.1_53, 1.1_301, 5.1_152, 4.1_46, 5.1_150| Ga0192377_1004_168, 1008_10–11, 1002_82, 1004_145 | TART1_1477, 1504, 2070, 2071, 2352
Salinity tolerance		
7	Glycine betaine transporter OpuD (IPR000060)	TR210_1348
3|5|6|7|8|9|10	Betaine binding ABC transporter protein (IPR000515)	Tpas_2814–2815|Tcol_1997|Tpat_1468|TR210_2767–2768,2770 | Ga0192364_3215_54–57 | PXZT01000008. _23–26| TART1_2694–96
1|2|5|7|8|9|10	Osmotically activated choline ABC transporter (IPR003439)	TES5_1206–1209|Tcol_773–776|TR210_342–345|Tflo_1131–1134|Ga0192364_2415_1215| PXZT01000003.2_54–57| TART1_266–69
2|3|6|7	Choline binding protein A (IPR010126)	TES5_1355|Tpas_1469|Tpat_1570|TR210_2363,1711,2104
1|2|3|5|6|8|9	Glycine betaine ABC transport system (IPR003439)	TES5_1660–1662|Tcol_1041–1043|Tpas_2619–21|Tpat_203–05|Tflo_1599–01 | Ga0192356_1653_23–25| PXZT01000006.2_75–77
Bacterial defence		
6|10	SNARE associated Golgi protein (IPR032816)	Tpat_1693,1825 | TART1_1950
4	Tetracycline resistance (IPR004638)	Tpal_1098,1664,1687
3	Toxin antidote HigA (IPR013430)	Tpas_511
3	Plasmid system killer (IPR007712)	Tpas_512
7	Bacteriocin class Iib (IPR010387, IPR029500)	Tflo_874,878–879
6	Cas9 (IPR028629)	Tpat_1430
1|2|6	Cas1 (IPR019855)	TES5_196|Tpat_1431|Tflo_184
6|10	Cas2 (IPR019199)	TES5_195|Tpat_1432| TART1_1189
1|2|3|7|10	Cas3 (IPR006935)	TES5_201|Tpas_1155|TR210_680|Tflo_179|TART1_0176
1|2|3|6	Cas5d (IPR021124)	TES5_200|Tpas_1156|TR210_679|Tflo_180
1|2|6|7	Casd1 (IPR010144)	TES5_199|Tpas_1157|TR210_675|Tflo_181
1|2|6|7	Csd2 (IPR006482)	TES5_198|Tpas_1158| TR210_677|Tflo_182
1|2|6|7	Csd4 (IPR022765)	Tpas_1159| TR210_676|Tflo_183

### Carbohydrate degradation by *Trichococcus* species

In general, *Trichococcus* species can utilise cellobiose, sucrose, maltose, and glucose [1–6]. Genes encoding proteins for the Embden-Meyerhof-Parnas (EMP) pathway and pentose phosphate pathway (PPP) were found in the genomes of the ten *Trichococcus* species analysed here. In addition, genes encoding proteins for the conversion of pyruvate to ethanol, acetate and lactate were found. This is consistent with the products (lactate, formate, acetate and ethanol) formed from glucose fermentation by the tested *Trichococcus* species (Table 2). Lactate was the main fermentation product, except in cultures of *T. patagoniensis*. The carbon fraction in lactate in cultures of *T. patagoniensis* was around 40% (calculated as carbon lactate/carbon all soluble products), while in other *Trichococcus* cultures lactate corresponded to 60–80% of the carbon detected in the products. Glucose fermentation by *T. patagoniensis* resulted in a relatively higher formate concentration, which is in agreement with the presence of a pyruvate formate-lyase in the genome of *T. patagoniensis* (Tpat_2317) and not in others. Ethanol yield in cultures of *T. patagoniensis* and *T. collinsii* was 0.2 and 0.1 mol_ethanol_/mol_consumed glucose_, respectively, which is higher than observed for the other *Trichococcus* species.

**Table 2:** Glucose (a) and glycerol (b) fermentation by *Trichococcus* species. Table shows substrate consumption and product generation (± standard deviation, triplicate assays), measured after 24 h for glucose fermentation experiments and after 40 h for glycerol fermentation experiments. Electron recovery was calculated based on substrate/product consumption/production and excludes electrons used for cellular growth

(a) Glucose Fermentation	Glucose consumed (mM)	Lactate (mM)	Formate (mM)	Acetate (mM)	Ethanol (mM)	Electron recovery (%)
*T. flocculiformis* (DSM 2094^T^)	19.1 ± 0.6	21.7 ± 2.1	6.9 ± 0.6	2.6 ± 0.3	3.9 ± 0.2	76.5 ± 1.5
Strain ES5 (DSM 23957)	19.6 ± 0.2	22.2 ± 0.7	7.5 ± 0.5	2.2 ± 0.1	4.1 ± 0.2	75.0 ± 0.5
*T. pasteurii* (DSM 2381^T^)	18.4 ± 1.0	23.8 ± 0.9	5.1 ± 0.4	1.5 ± 0.0	1.9 ± 0.7	77.2 ± 1.3
*T. palustris* (DSM 9172^T^)	19.2 ± 0.4	16.2 ± 0.9	12.6 ± 0.4	4.9 ± 0.4	6.6 ± 0.2	74.1 ± 0.7
*T. collinsii* (DSM 14526^T^)	13.1 ± 0.6	20.2 ± 0.6	3.3 ± 0.3	0.6 ± 0.2	1.1 ± 0.1	90.4 ± 0.8
*T. patagoniensis* (DSM 18806^T^)	19.1 ± 0.9	11.4 ± 1.0	18.2 ± 0.9	6.8 ± 0.3	9.0 ± 0.3	75.1 ± 1.0
*T. ilyis* (DSM 22150^T^)	18.9 ± 0.6	19.8 ± 0.9	8.8 ± 0.5	3.2 ± 0.3	4.6 ± 0.3	75.5 ± 0.8
(b) Glycerol Fermentation	Glycerol consumed (mM)	Lactate (mM)	Formate (mM)	Acetate (mM)	1,3-PDO (mM)	Electron recovery (%)
*T. pasteurii* (DSM 2381^T^)	18.5	0.5 ± 0.1	0.9 ± 0.5	3.6 ± 0.7	13.8 ± 0.2	99.4 ± 0.6
Strain ES5 (DSM 23957)	19.0	0.5 ± 0.0	2.3 ± 0.2	4.5 ± 0.2	12.3 ± 0.1	90.7 ± 0.1

*T. pasteurii* and *Trichococcus* strain ES5 can ferment glycerol. The most abundant product from glycerol fermentation by *T. pasteurii* and *Trichococcus* strain ES5 is 1,3-propanediol (1,3-PDO), which represents about 70–80% of the total carbon detected in products (Table 2). The genomes of these species contain an identical large operon (17 genes organized in identical fashion and with 100% sequence identity), which is involved in glycerol conversion (Table 1). This operon is absent in the other eight studied *Trichococcus* species that cannot degrade glycerol. Two of the genes in this operon are essential for glycerol conversion to 1,3-PDO: glycerol dehydratase (alpha, beta and gamma subunits) and 1,3-propanediol dehydrogenase. Additional genes in the operon encode for: a glycerol uptake facilitator, a glycerol dehydratase activator (involved in the activation of glycerol dehydratase), and cobalamin adenosyltransferase which is involved in the conversion of cobalamin (vitamin B12) to its coenzyme form, adenosylcobalamin (glycerol dehydratase requires vitamin B12 as a binding co-factor [21]).

*T. collinsii* has unique domains related to alginate utilisation and encodes three alginate lyases (Table 1). In vitro testing confirmed that *T. collinsii* utilises alginate (optical density increase of about 0.2 after 72 h incubation).

In the genome of *T. patagoniensis*, 17 homologous domains of glycoside hydrolases family 1 (includes e.g. glucosidases, galactosidases and hydrolases) were identified, but they all belong to genes encoding hypothetical proteins (Table 1). Metal-dependent hydrolases were identified with 12 homologous genes in the genome of *T. patagoniensis*. In addition, two copies of the gene encoding for extracellular endo-alpha-(1- > 5)-L-arabinanase are present in the genome. This enzyme catalyses the degradation of arabinan and it is an important enzyme in the degradation of the plant cell wall. To confirm the protein domains prediction, growth of *T. patagoniensis* on arabinan was tested in vitro. *T. patagoniensis* could utilise and grow on arabinan (OD of 0.25 ± 0.02 after 96 h incubation).

### Growth of *Trichococcus* species at low temperature

Six cold shock domains (CSD) (IPR011129) were encoded in all *Trichococcus* genomes (Table 1). One additional CSD was encoded in the genomes of *T. palustris* and *T. ilyis*. The conserved CSDs in *Trichococcus* species were neighbouring genes encoding for domains of the cold-shock DNA-binding site (IPR002059), the nucleic acid-binding OB-fold (IPR012340) and the cold-shock conserved site (IPR019844). One of the CSD is part of a cold shock protein (Table 1), which contains additional domains likely involved in the transcription and regulation of the cold protection mechanisms: ATPase F1 nucleotide-binding (IPR000194), AAA+ ATPase (IPR003593), transcription termination factor Rho (IPR004665), rho termination factor N-terminal (IPR011112), rho termination factor RNA-binding domain (IPR011113), nucleic acid-binding OB-fold domain (IPR012340) and P-loop containing nucleoside triphosphate hydrolase domain (IPR027417). Genomes of twenty-two LAB species closely related to *Trichococcus* species were analysed for CSDs (complete list of LAB species in Additional file 1: Table S1). A similar cold shock protein to the one encoded in the genomes of *Trichococus* species was identified in the twenty-two genomes of LAB species, but only seven LAB species contain six to eight additional CSD (*Carnobacterium mobile*, *C. pleistocenium, C. jeotgali, C. inhibens*, *C. funditum*, *C. maltaromaticum, C. alterfunditum*)*.*

Overall, *Trichococcus* species can grow at temperatures lower than their optimum growth temperature (25–30 °C) [1–6]. Only four of the *Trichococcus* species tested in this study were able to grow at 0 °C (on glucose, and over 45 days of incubation): *T. pasteurii, T. collinsii, T. patagoniensis* and *Trichococcus* strain ES5 (Additional file 4: Figure S2)*.* At 0 °C, *T. patagoniensis* and *T. palustris* had a lag phase of eight days, whereas growth of *T. collinsii* and *Trichococcus* strain ES5 was only observed after 23 days of incubation. The recently described *T. shcherbakoviae* is also able to grow at freezing temperatures [4].

### Resistance of *Trichococcus* to high salinity

Functional genome analysis resulted in the identification of protein domains related to osmoregulation in all the *Trichococcus* species, except in *T. palustris* (Table 1). Multiple domains related to glycine and betaine transport systems could be identified*.* These transport systems are important for living at high salinity because, during osmotic pressure, bacterial cells can increase the concentration of uncharged osmoprotectants (glycine, betaine) in the cytoplasm [22, 23]. In addition, choline transporters were also identified. Glycine and betaine can be formed from choline [24].

Salinity tolerance for the different *Trichococcus* species was tested. Only *T. palustris* was sensitive to salinity, and growth was inhibited at 2% NaCl (Additional file 4: Figure S3). All the other tested strains could grow in media with a NaCl concentration of 2%. At 4% salinity and after 6 days, growth was observed for only four of the tested bacteria: *T. pasteurii, T. patagoniensis, T. flocculiformis*, and *Trichococcus* strain ES5*.* After ten days, weak growth was observed at 6% NaCl for *T. patagoniensis, T. pasteurii* and *Trichococcus* strain ES5 (Additional file 4: Figure S3). *T. paludicola* and *T. alkaliphilus* were previously observed to tolerate NaCl concentrations up to 4.5% [6].

### CRISPR and antibiotic resistance genes in *Trichococcus* species

Recent studies support the effective defence of the CRISPR system in bacteria against viral threats [25]. The CRISPR system contains Cas genes which introduce double strand breaks in foreign DNA in the cells. Cas genes were present in *T. flocculiformis*, *T. pasteurii, T. patagoniensis, T. ilyis,* and *Trichococcus* strain ES5 (Table 1). The CRISPR system in *T. patagoniensis* can be classified as Cas2, type II-C, while the other studied *Trichococcus* species encode the class 1 type I-C CRISPR system. Several spacer sequences (i.e. foreign nucleic acid sequences merged in the genome by CRISPR systems) were found in the genomes *Trichococcus* species: *T. pasteurii* (115 spacer sequences), *T. patagoniensis* (88 spacer sequences), *Trichococcus* strain ES5 (82 spacer sequences), *T. ilyis* (80 spacer sequences), *T. fluccoliformis* (27 spacer sequences). The alignment of the spacers sequences from the analysed *Trichococcus* species resulted in low similarity, likely not containing common foreign DNA.

Alternative defense mechanisms were also found (Table 1). The domain of SNARE associated Golgi protein was encoded in the genomes of *T. patagoniensis* and *T. shcherbakoviae*. SNARE proteins can be used for promoting or blocking membrane fusion and act especially against eukaryotic cells [26]. *T. palustris* contains genes encoding for tetracycline resistance proteins (Table 1), which were not found in the genomes of the other *Trichococcus* species. Agar plates containing *Clostridium* medium and increasing concentrations of tetracycline (0.016–256 μg/mL) were used to test resistance to this antibiotic. *T. palustris* could grow in plates containing 4 μg/mL, whereas *T. ilyis* and *T. palustris* did not tolerate tetracycline at this concentration. Genes encoding a toxin antidote protein HigA and a plasmid system killer were found in *T. pasteurii* (Table 1). The two genes are associated with bacterial toxin-antitoxin (TA) proteins and regulate the tolerance of the cells at environment and chemical stress [27]. The genome of *T. flocculiformis* contains three homologous genes for the domain bacteriocin class IIb, which is commonly associated with growth inhibition of several microorganisms [28].

### Comparison of *Trichococcus *strain ES5 and *T.flocculiformis*

**Fig. 3:**
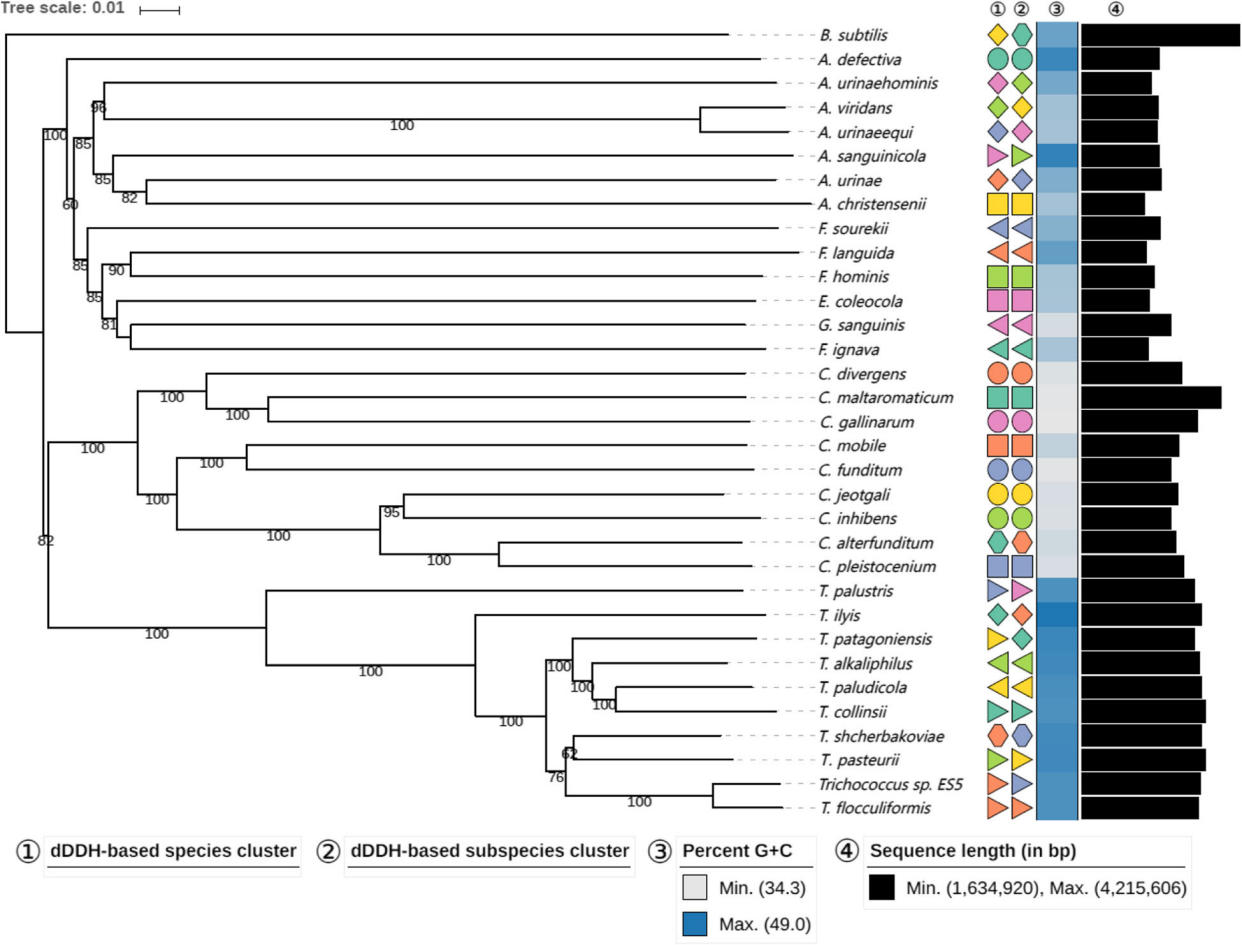
Genome-based phylogenomic analysis of *Trichococcus* species restricted to coding regions. Tree inferred with FastME 2.1.4 from Genome Blast Distance Phylogeny GBDP distances calculated from the ten *Trichococcus* species, 22 LAB species and *B. subtilis* genome sequences. The branch lengths are scaled in terms of GBDP distance. The numbers above branches are GBDP pseudo-bootstrap support values from 100 replications, with an average branch support of 88%. Leaf labels are further annotated by their affiliation to species (①, identical symbol shape and color indicate same species clade) and subspecies (②, identical symbol shape and color indicate same subspecies clade) clusters as well as their genomic G + C content ③ and their overall genome sequence length ④

*Trichococcus* strain ES5 was previously isolated by van Gelder et al. [12]. Based on 16S rRNA gene comparison, strain ES5 was phylogenetically closely related to *T. flocculiformis* (99%). However, it is known that *Trichococcus* species have a highly conserved 16S rRNA gene and a correct taxonomic affiliation demands DNA-DNA hybridization [3, 4, 6]. Digital DNA-DNA hybridization (dDDH) between strain ES5 and *T. flocculiformis* is 71%, with a confidence interval between [68.0–73.9%] (Additional file 5). This value is just above the 70% cut-off value generally recommended for species differentiation [7]. Furthermore, it is below the 79% cut-off value for subspecies delineation [29]. Average Nucleotide Identity (ANI) between strain ES5 and *T. flocculiformis* is 95.9%, which is above the cut-off value of 95% [8]. Based on these results strain ES5 is a *T. flocculiformis* strain (Fig. 3; Additional file 5). Nevertheless, strain ES5 has unique physiological properties that are not observed in the type strain, such as the ability to ferment glycerol and an apparent higher tolerance to salinity (could grow at 6% NaCl).

## Discussion

The comparative analysis of *Trichococcus* species described here served two purposes. First, it allowed to identify and predict novel physiological traits within the genus *Trichococcus* species. Second, a proper taxonomic position of the several analysed *Trichococcus* strains could be made.

### Taxonomic classification of *Trichococcus* species

The 16S rRNA gene is commonly used for taxonomic classification. However, this gene of *Trichoccocus* species is highly conserved and thus it cannot be used for taxonomical classification at species level. Therefore, assigning a novel *Trichococcus* strain to a certain species is more challenging than in other genera. As an example, *T. patagoniensis* and *T. collinsii* have a 100% similar 16S rRNA gene sequence and additional tests were needed to show that they belonged to different species [5]. *Trichococcus* is not the only genus with conserved 16S rRNA genes. Other examples are e.g. *Edwardsiella*, *Clostridium* and *Mycobacterium* [30–32]. Novel omics approaches are helpful in this respect. Previously, the description of two new *Trichococcus* species (*T. ilyis* and *T. shcherbakovii*) was done by complementing 16S rRNA gene analysis with genome-based dDDH [3, 4]. A similar approach was applied for the assignment of *T. paludicola* and *T. alkaliphilus* [6], and here we could show that the previously isolate strain ES5 is a *T. flocculiformis* strain, though some of its physiological properties, such as the ability to grow with glycerol, were different from the type strain. It can be concluded that the use of genomics information (such as dDDH and ANI) can help the taxonomical clustering of novel species in the genus of *Trichococcus* and in other genera as an efficient and accurate approach.

### Extended substrate use of *Trichococcus* species

The genome-guided approach that was followed in this study shed light on the physiological similarities and differences of *Trichococcus* species. The presence of genes coding for protein domains related to carbohydrate conversion confirmed the use of previously tested sugar-substrates. Importantly, novel growth substrates can be identified by genomics analysis, and further tested in defined experimental approaches. Usually, laborious substrate tests, based on a somehow random selection, are needed to define which substrates a newly isolated bacterium can use. However, genome analysis can aid in the selection of the most likely substrates to be converted by a specific bacterium. Some members of the genus *Trichococcus* (*T. pasteurii* and strain ES5) possess an operon of 17 genes involved in glycerol degradation and 1,3-PDO production and these strains were able to ferment glycerol and produce 1,3-propanediol (1,3-PDO) as a main fermentation product. The strains tested that lacked that operon were not able to ferment glycerol. For both strains in vitro assays showed glycerol fermentation and 1,3-PDO production. Similarly, we identified genes involved in alginate degradation in *T. collinsii* and involved in arabinan degradation in *T. patagoniensis*. These two strains tested positive for growth on the respective substrates. It should be noted that when dedicated genes are detected, growth with that particular substrate is not always observed and to ascertain this experimental testing is necessary. For example, genes involved in degradation of tagatose, starch and L-sorbose were present in the genome of *T. ilyis*, but in vitro bacterial growth with these compounds was not observed [3].

### Growth of *Trichoccus* species at low temperature

Psychrophylic and psychrotolerant microorganisms, due to the extreme environmental conditions, need to adapt and obtain protection mechanisms [33]. All *Trichococcus* species possess a high number of cold shock domains (CSD), genes related with a psychrotolerant phenotype. However, only five species can grow at 0 °C (i.e. *T. pasteurii, T. collinsii, T. patagoniensis* and *Trichococcus* strain ES5, and *T. shcherbakoviae*). For comparison of CSD, we included 20 lactic acid bacteria (LAB), belonging to the genera of *Carnobacterium* and *Aerococccus.* Species of these genera that had been isolated from low temperature had multiple CSDs that resembled those in *Trichococcus* species. Other possible bacterial adaptation to low temperature is the production of cryoprotectant exopolymeric substances (EPS), which can surround the cells and create a protective layer against cold [34, 35]. A mucoid substance has been observed in *T. patagoniensis* [5], which is likely related to its capacity to grow at 0 °C. Antifreezing compounds are of potential interest for applications in food bioindustry, agriculture (e.g. incorporation in fertilizers for increasing cold resistance of plants), and medicine (cryopreservation of cells).

## Conclusion

Genome-guided characterisation of *Trichococcus* species resulted in the discovery of novel functional traits within this genus. This approach revealed a large operon that encodes the necessary enzymes for the production of 1,3-PDO from glycerol, which is present in *T. pasteuri* and *Trichococcus* strain ES5. It also enabled the identification of genes associated with the degradation of complex molecules, such as alginate and arabinan, in the genomes of some of the analysed *Trichococcus* species. These metabolic traits of *Trichococcus* species may set them as possible candidates in biotechnological processes related to the degradation or production of these compounds. Their robust phenotype, ability to grow at low temperature and high salinity, may foster versatile applications (e.g. conversion of organic compounds in high-salinity wastewaters to added-value products). The CRISPR system and the unique defence mechanisms in *Trichococcus* species provide them against viral attacks, which can confer them higher robustness for industrial applications.

## Materials and methods

### Source of genomes

The genome of *Trichococcus* strain ES5 (DSM 23957) was sequenced at the Joint Genome Institute from the US Department of Energy (JGI-DOE) (Walnut Creek, CA) using an Illumina HiSeq2000 platform (Illumina Inc., San Diego, CA). This genome (11,259,926 reads and 151 bp read length) was assembled and annotated as described previously [3]. All the publicly available genome sequences of *Trichococcus* species, i.e. *T. flocculiformis* (DSM 2094^T^), *T. pasteurii* (DSM 2381^T^), *T. palustris* (DSM 9172^T^), *T. collinsii* (DSM 14526^T^), *T. patagoniensis* (DSM 18806^T^), *T. ilyis* (DSM 22150^T^)*, T. paludicola* (DSM 104691^T^)*, T. alkaliphilus* (DSM 104692^T^)*,* and *T. shcherbakoviae* (DSM 107162^T^), were obtained from the NCBI Assembly Database [36]. The same database was used to retrieve sequences of twenty-two related lactic acid bacteria (LAB) to *Trichococcus* species and *Bacillus subtilis* (outgroup species), for taxonomic hierarchical analysis. A complete list of the LAB used in the comparison is included in (Additional file 1: Table S1).

### Functional analysis and genome annotation

Genomes from *Trichococcus* species (ten), LAB species (twenty-two), and *B. subtilis* were annotated using the pipeline of Semantic Annotation Platform with Provenance (SAPP) that includes Prodigal v2.6 for predicting coding gene sequences [19, 37]. *T. paludicola* and *T. alkaliphilus* locus tags were based on Prodigal v2.6 prediction (*T. paludicola*: Ga019, *T. alkaliphilus:* PXZT) for comparison purposes. Functional genome analysis was based on protein Hidden Markov Model domains (HMM) generated by InterProScan v5.17–56.0 based on Pfam domains (−-app pfam) [38–40]. InterPro protein domains matrix was generated for all the *Trichococcus*, selected LAB, and *B. subtilis*. *B. subtilis* was used as an outgroup for the study and was not included in the core and unique protein domain analysis. Core protein domains (present in all compared genomes) and unique protein domains (present in only one of the analysed genomes) were identified. The presence/absence matrix of protein domains from all species was converted to distances by using the dice coefficient method and a neighbour-joining tree was generated. For functional protein domain clustering, the analysis was performed in R and confirmed with DARwin v6.0 [20]. In addition, 16S rRNA gene sequences were extracted from the genomes and aligned using the software CLC Main Workbench v8.0 (CLC Bio, Aarhus, Denmark). A neighbour-joining tree was constructed based on 16S rRNA gene sequences.

### Whole-genome based analyses

All pairs of strains were compared using the Genome-to-Genome Distance Calculator 2.1 (GGDC; https://ggdc.dsmz.de) under recommended settings [7] and pairwise digital DNA-DNA hybridisation values (dDDH) were inferred accordingly. Afterwards, the distance matrix was subjected to a clustering using established thresholds for delineating species [7] as well as subspecies [29]. Clustering was done using the OPTSIL clustering program [41].

A genome sequence-based phylogenetic analysis based on the coding regions was conducted using the latest version of the Genome-BLAST Distance Phylogeny (GBDP) method as previously described [42]. Briefly, BLAST+ [43] was used as a local alignment tool and distance calculations were done under recommended settings (greedy-with-trimming algorithm, formula d_5_, e-value filter 10^− 8^). A calculation of 100 replicate distances for pseudo-bootstrap support was included. Finally, a balanced minimum evolution tree was inferred using FastME v2.1.4 with SPR post processing [44]. A similar approach was used for the reconstruction of replicate trees and branch support was subsequently mapped onto the tree. Finally, exchanged genomic syntenies were defined with Sibelia v3.0.6 [45] using default parameters, and visualised in circular graph by Circos v0.69 [46].

### Microbial growth tests

Growth experiments were conducted with anaerobic basal medium prepared as previously described [47]. 45 mL of medium were dispensed in 120 mL serum bottles, which were sealed with rubber stoppers and aluminium caps. Bottles’ headspace was flushed with N_2_/CO_2_ (80/20 v/v) to a final pressure of 1.5 bar. After autoclaving, and before inoculation, medium was supplemented with 0.5 mL of salts solution and 2.5 mL of bicarbonate solution [47]. Yeast extract was added to the medium at a concentration of 0.1 g/L. Substrates were added to the medium from sterile stock solutions. Glucose and glycerol growth assays were done with an initial substrate concentration of 20 mM. Degradation of alginate was tested with a concentration of 5 mM and arabinan (sugar beet, Ara:Gal:Rha:GalUA = 88:3:2:7) with a concentration of 0.4% (v/v). Incubations were in the dark, without stirring and at 30 °C (unless stated otherwise). All tests were done in triplicate. Controls without substrate and blanks without inoculation were also performed.

### Antibiotic resistance tests

Antibiotic resistance tests for tetracycline were performed in plates with rich *Clostridium* medium (Fisher Scientific, PA) and 1% agar. Minimum inhibitory concentration (MIC) tetracycline test stripes were used with a test range of 0.016–256 μg/mL (Liofilchem, Roseto degli Abruzzi, Italy). Plates were incubated at 30 °C in anaerobic containers.

### Psychrotolerance and salinity test

Temperature and salinity tests were performed using 20 mM of glucose as substrate and using the anaerobic basal medium previously described [47]. Growth of all members of *Trichococcus* genus was tested at 0 °C and monitored for 45 days. For salinity tolerance experiments, sodium chloride was used at concentrations of 2, 4, 6, 8, 10% (w/v). Growth of *Trichococcus* species at different salinities was monitored for ten days.

### Analytical measurements

Growth was quantified by optical density (OD 600 nm), measured in a spectrometer (Hitachi U-1500, Labstuff, The Netherlands). Soluble metabolites, such as glucose, glycerol, 1,3-PDO, lactate, ethanol, acetate and formate were measured with Thermo Electron HPLC system equipped with an Agilent Metacarb 67H column (Thermo, Waltham, MA), which had as mobile phase sulphuric acid (5 mM) at a flow rate of 0.8 mL min^− 1^ and temperature at 45 °C.

## Supplementary information

**Additional file 1.** General genomic information of all species used for in silico analysis in this study.

**Additional file 2.** SAPP-generated protein domain matrix. XLSX 628 kb.

**Additional file 3. **Common protein domains in *Trichococcus* strains. XLSX 28 kb.

**Additional file 4. **Growth curves of *Trichococcus* species at 0° and at different salinities (0–10% NaCl (w/v)). DOCX 155 kb.

**Additional file 5. **Outputs of dDDH and ANI analyses comparing strain ES5 and *T. flocculiformis*. XLSX 22 kb.

## Data Availability

The data from this study are available in the manuscript and additional file. Genomic data are deposited in public databases (accession numbers are provided in Additional File 1: Table S1). The genomic sequence data of *Trichococcus* strain ES5 that supports the findings of this study have been deposited in GenBank with accession codes GCA_900067165.1, GCF_900067165.1.

## Abbreviations

1,3-PDO: 1,3-Propanediol
CSD: Cold Shock Domains
dDDH: Digital DNA-DNA Hybridisation
EMP: Embden-Meyerhof-Parnas pathway
GBDP: Genome-BLAST Distance Phylogeny
GGDC: Genome-to-Genome Distance Calculator
HMM: Hidden Markov Model domains
LAB: Lactic Acid Bacteria
OD: Optical Density
PPP: Pentose Phosphate Pathway
SAPP: Semantic Annotation Platform with Provenance
TA: Toxin-Antitoxin
