# Supplementary material for: Genome-guided analysis allows the identification of novel physiological traits in Trichococcus species
Source: BMC Genomics. 2020 Jan 8;21:24. doi: 10.1186/s12864-019-6410-x (PMC6950789; doi:10.1186/s12864-019-6410-x)
Supplement: Supplementary file 1 — Additional file 1. General genomic information of all species used for in silico analysis in this study. [file 12864_2019_6410_MOESM1_ESM.docx]

**Genome-guided analyses allow the identification of novel physiological traits in *Trichococcus* species**

Nikolaos Strepis^1,2^, Henry D. Naranjo^1^, Jan Meier-Kolthoff^3^, Markus Göker^3^, Nicole Shapiro^4^, Nikos Kyrpides^4^, Hans-Peter Klenk^3,5^, Peter J. Schaap^2^, Alfons j. M. Stams^1,6^, Diana Z. Sousa^1*^

^1^ Laboratory of Microbiology, Wageningen University & Research, Stippeneng 4, 6708 WE Wageningen, The Netherlands

^2^ Laboratory of Systems and Synthetic Biology, Wageningen University & Research, Stippeneng 4, 6708 WE Wageningen, The Netherlands

^3^ Leibniz Institute DSMZ German Collection of Microorganisms and Cell Cultures, Inhoffenstraße 7B, 38124 Braunschweig, Germany

^4^ DOE Joint Genome Institute, 2800 Mitchell Drive 100, CA 94598 Walnut Creek, CA USA

^5^ School of Biology, Newcastle University, Ridley Building 2, NE1 7RU Newcastle, UK

^6^ Centre of Biological Engineering, University of Minho, Campus de Gualtar, 4710-057 Braga, Portugal

*Corresponding author: Diana Z. Sousa, e-mail: [diana.sousa@wur.nl](mailto:diana.sousa@wur.nl)

**ADDITIONAL FILE 1**

**Identification of bacterial species included in the genome comparison in this study**

**Table S1. Information on the microorganisms used in the genomic comparison presented in this study.** Source of isolation was obtained from the recorded metadata in the genome publication and/or publication of species description. Contigs are stated as the number of contigs submitted in the public databases for each species genome. GC content is represented in percentage (%) and was obtained from the genomic sequence. Number of domains includes the all domains identified from the InteProScan analysis. Pathogenicity is reported as stated in the public records for each species. GenBank Assembly Accession ID is based on NCBI assembly format of GCA_ID_Number.

| **Species** | **Source of isolation** | **DSM ID** | **Genome size (bp)** | **Number**  **of Contigs** | **% GC** | **Number of domains** | **Pathogenic** | **GenBank assembly accession ID (GCA_)*** |
| --- | --- | --- | --- | --- | --- | --- | --- | --- |
| *Trichococcus flocculiformis* | Activated sludge | 2094 | 3,23895 | 91 | 44 | 3386 | No | 900067465.1 |
| *Trichococcus strain ES5* | Anaerobic granular sludge | 23957 | 3194513 | 50 | 44 | 3474 | No | 900067165.1 |
| *Trichococcus pasteurii* | Septic pit | 2381 | 3288204 | 21 | 45 | 3657 | No | 900070335.1 |
| *Trichococcus collinsii* | Hydrocarbon spilt site | 14526 | 3343507 | 6 | 44 | 3678 | No | 900067155.1 |
| *Trichococcus palustris* | Swamp | 9172 | 3004925 | 17 | 46 | 3270 | No | 900067125.1 |
| *Trichococcus patagoniensis* | Guano from penguin Patagonia | 18806 | 3017565 | 38 | 47 | 3524 | No | 003051085.1 |
| *Trichococcus ilyis* | Sulphate reducing bioreactor | 22150 | 3194513 | 44 | 48 | 3499 | No | 900067235.1 |
| *Trichococcus paludicola* | High elevation wetland, Tibetan Plateau | 104691 | 1935222 | 5 | 46 | 2305 | No | 003052295.1 |
| *Trichococcus alkaliphilus* | High elevation wetland, Tibetan Plateau | 104692 | 2964741 | 25 | 46 | 3401 | No | 003008695.1 |
| *Trichococcus shcherbakoviae* | Granulated biomass of psychrophilic bioreactor | 107162 | 3137223 | 51 | 46 | 3558 | No | 900416805.1 |
| *Aerococcus sanguinicola* | Human blood | 14282 | 2033849 | 1 | 47 | 6523 | Yes | 001543145.1 |
| *Aerococcus christensenii* | Human genitals | 15819 | 1655357 | 46 | 39 | 3826 | Yes | 001543105.1 |
| *Aerococcus urinae* | Urinary tract, infection | 7446 | 1974262 | 1 | 42 | 4943 | Yes | 001543175.1 |
| *Aerococcus urinaeequi* | Human peritoneal-related ascites | 20341 | 2017038 | 9 | 39 | 4921 | Yes | 001518795.1 |
| *Aerococcus urinaehominis* | Human urinary tract | 15634 | 1854293 | 1 | 42 | 4318 | Yes | 001543245.1 |
| *Aerococcus viridans* | Air and dust | 20340 | 2018981 | 31 | 39 | 4883 | Yes | 001543285.1 |
| *Abiotrophia*  *defective* | Human intestine | 9849 | 2068984 | 4 | 46 | 4621 | Yes | 000160075.2 |
| *Carnobacterium alterfunditum* | Ace Lake Antarctica | 5972 | 2506249 | 1 | 36 | 5986 | No | 000744115.1 |
| *Carnobacterium divergens* | Gut Atlantic Salmon | 20623 | 2651963 | 1 | 35 | 6180 | No | 000744255.1 |
| *Carnobacterium funditum* | Ace Lake, Antarctica | 5971 | 2362574 | 1 | 35 | 5529 | No | 000744185.1 |
| *Carnobacterium gallinarum* | Hindgut chambers, Atlantic cod | 4847 | 3081008 | 1 | 34 | 6089 | No | 000744375.1 |
| *Carnobacterium inhibens* | Gut Atlantic Salmon | 13024 | 2381355 | 1 | 35 | 5747 | No | 000493735.1 |
| *Carnobacterium jeotgali* | Korean traditional fermented food | 23388 | 2549728 | 12 | 35 | 6029 | No | 000745125.1 |
| *Carnobacterium maltaromaticum* | Fresh pork | 20344 | 3696047 | 1 | 34 | 7976 | Yes | 000317975.2 |
| *Carnobacterium mobile* | Irradiated chicken meat | 4848 | 2632867 | 6 | 37 | 5912 | No | 000744825.1 |
| *Carnobacterium pleistocenium* | Permafrost of the Fox Tunnel Alaska | 17715 | 2695901 | 1 | 35 | 6334 | No | 000744285.1 |
| *Eremococcus coleocola* | Reproductive tract of horse | 15696 | 1795927 | 21 | 38 | 4350 | Yes | 000428865.1 |
| *Facklamia hominis* | Human abscess on buttocks | - | 1921344 | 1 | 38 | 4300 | Yes | 000301035.1 |
| *Facklamia ignava* | Human clinical specimens | - | 1788753 | 6 | 38 | 4166 | Yes | 000301055.1 |
| *Facklamia languida* | Human clinical specimens | - | 1739240 | 1 | 43 | 3926 | Yes | 000245795.1 |
| *Facklamia sourekii* | Human sources | 104271 | 2092896 | 48 | 41 | 4917 | Yes | 000245795.1 |
| *Globicatella sanguinis* | Cerebrospinal fluid | 7447 | 2356976 | 256 | 35 | 5259 | Yes | 001552295.1 |
| *Bacillus subtilis* | Soil | 347 | 4215606 | 1 | 44 | 4387 | No | 000009045.1 |

* GenBank accession numbers are given for the NCBI ‘Assembly Reports’; these reports are publicly available in <https://www.ncbi.nlm.nih.gov/assembly/>, by searching GCA_assembling-accession-number (*e.g.* GCA_900067465.1).

**Completeness assessment for genomic data quality control of *Trichococcus* species included in this study**


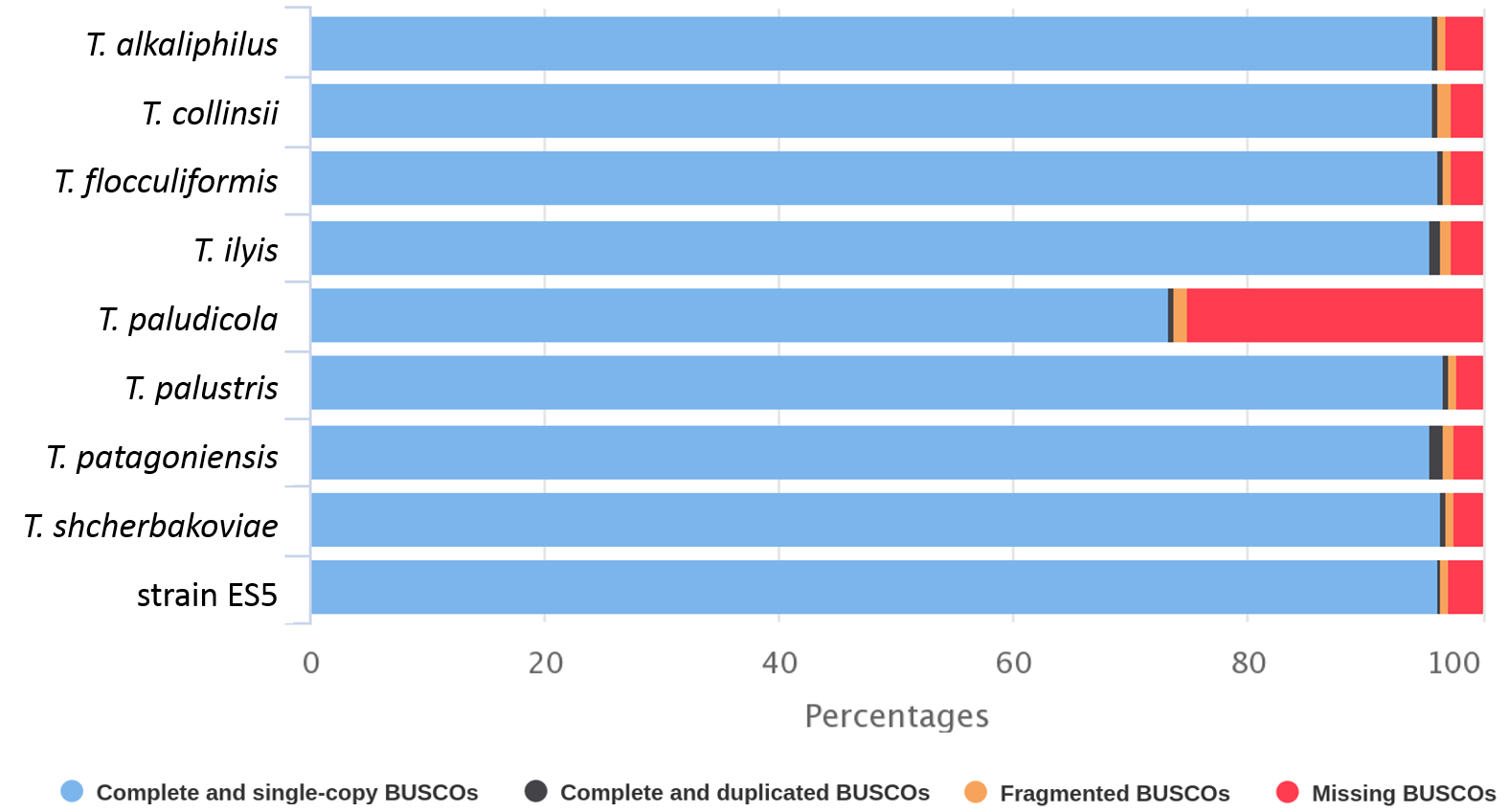


**Figure S1. BUSCO (“Benchmarking Universal Single-Copy Ortholog”) completeness assessment for genomic data quality control of *Trichococcus* species analysed in this study.** Genome completeness using the Benchmarking Universal Single-Copy Orthologs (BUSCO, RRID:SCR_015008; version 3.0) software [1]. Graph was generated using MultiQC [2].

[1] Simão FA, Waterhouse RM, Ioannidis P, Kriventseva EV, Zdobnov EM: BUSCO: assessing genome assembly and annotation completeness with single-copy orthologs. *Bioinformatics* 2015, 31(19):3210–3212, <https://doi.org/10.1093/bioinformatics/btv351>

[2] Ewels P, Magnusson M, Lundin S, Käller M: MultiQC: summarize analysis results for multiple tools and samples in a single report. Bioinformatics 2016, 32(19):3047–3048, <https://doi.org/10.1093/bioinformatics/btw354>.
