# Supplementary material for: Genome-guided analysis allows the identification of novel physiological traits in Trichococcus species
Source: BMC Genomics. 2020 Jan 8;21:24. doi: 10.1186/s12864-019-6410-x (PMC6950789; doi:10.1186/s12864-019-6410-x)
Supplement: Supplementary file 4 — Additional file 4. Growth curves of Trichococcus species at 0° and at different salinities (0–10% NaCl (w/v)). DOCX 155 kb. [file 12864_2019_6410_MOESM4_ESM.docx]

**Genome-guided analyses allow the identification of novel physiological traits in *Trichococcus* species**

Nikolaos Strepis^1,2^, Henry D. Naranjo^1^, Jan Meier-Kolthoff^3^, Markus Göker^3^, Nicole Shapiro^4^, Nikos Kyrpides^4^, Hans-Peter Klenk^3,5^, Peter J. Schaap^2^, Alfons j. M. Stams^1,6^, Diana Z. Sousa^1*^

^1^ Laboratory of Microbiology, Wageningen University & Research, Stippeneng 4, 6708 WE Wageningen, The Netherlands

^2^ Laboratory of Systems and Synthetic Biology, Wageningen University & Research, Stippeneng 4, 6708 WE Wageningen, The Netherlands

^3^ Leibniz Institute DSMZ German Collection of Microorganisms and Cell Cultures, Inhoffenstraße 7B, 38124 Braunschweig, Germany

^4^ DOE Joint Genome Institute, 2800 Mitchell Drive 100, CA 94598 Walnut Creek, CA USA

^5^ School of Biology, Newcastle University, Ridley Building 2, NE1 7RU Newcastle, UK

^6^ Centre of Biological Engineering, University of Minho, Campus de Gualtar, 4710-057 Braga, Portugal

*Corresponding author: Diana Z. Sousa, e-mail: [diana.sousa@wur.nl](mailto:diana.sousa@wur.nl)

**ADDITIONAL FILE 4**

**
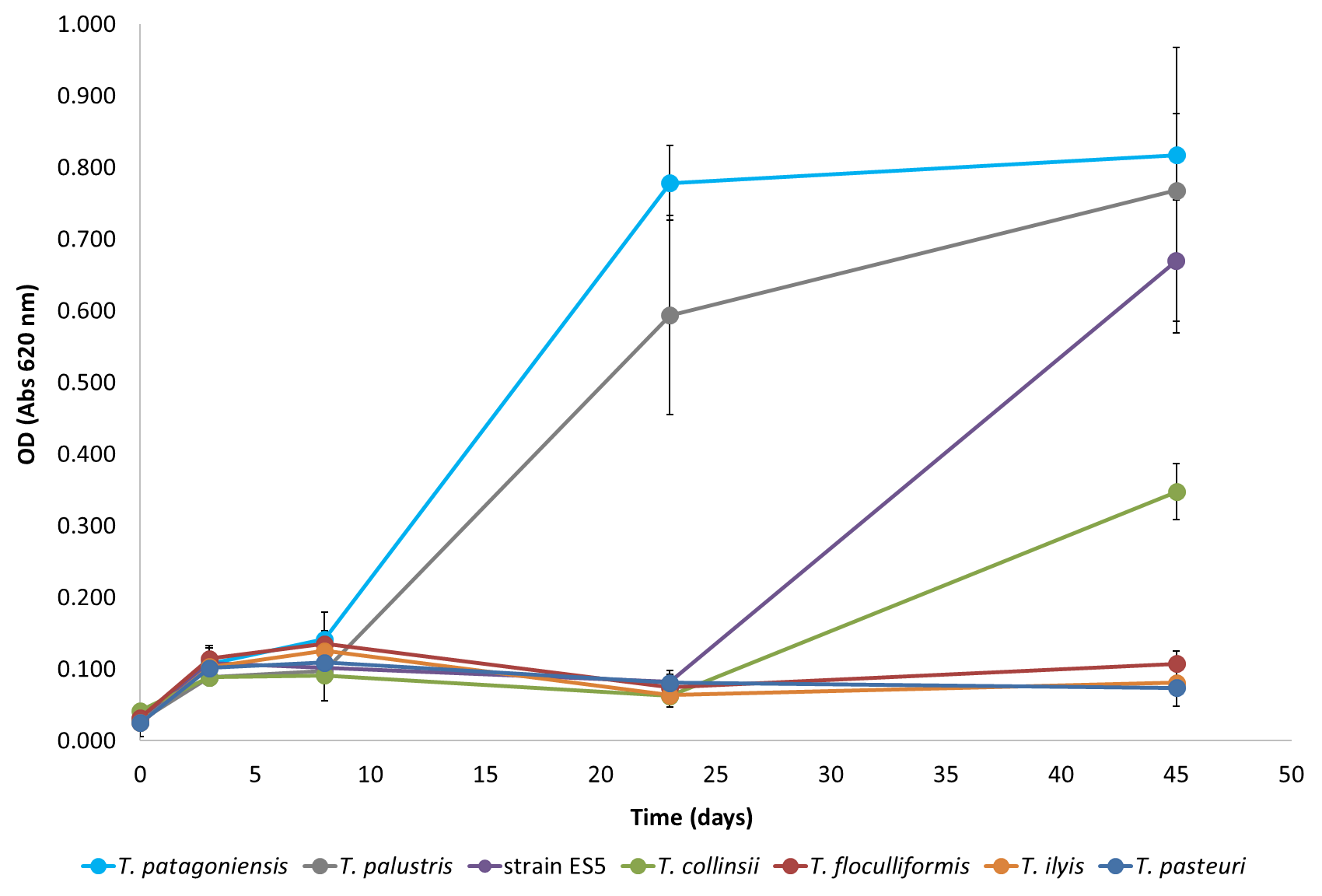
**

**Figure S2.** Growth of *Trichococcus* species at 0 ^o^C.


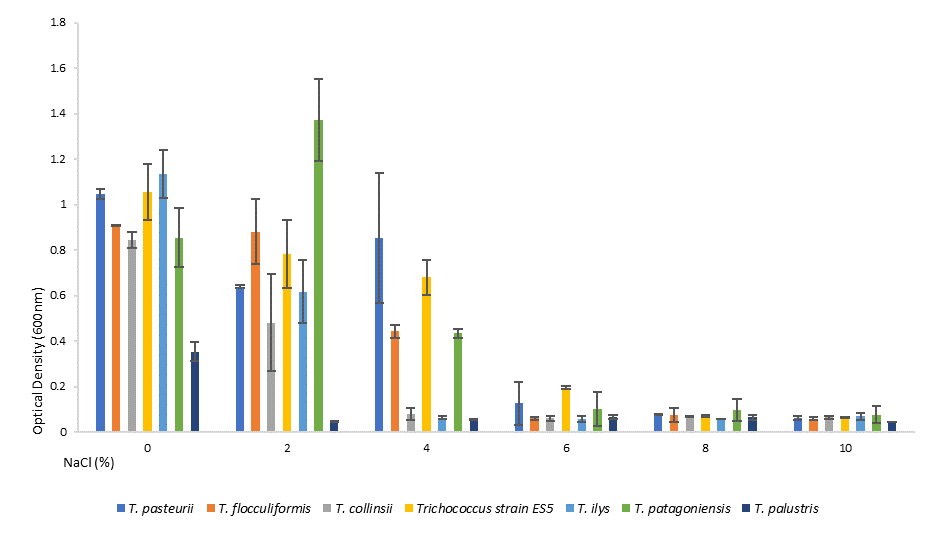


**Figure S3.** Growth of *Trichococcus* species at different salinity (% NaCl (w/v)).
